# Cellular analysis of bronchoalveolar lavage fluid to narrow differential diagnosis of checkpoint inhibitor-related pneumonitis in metastatic melanoma

**DOI:** 10.1186/s12967-020-02650-z

**Published:** 2020-12-10

**Authors:** Sabino Strippoli, Livia Fucci, Antonio Negri, Daniela Putignano, Marco Luigi Cisternino, Gaetano Napoli, Ruggiero Filannino, Ivana De Risi, Angela Monica Sciacovelli, Michele Guida

**Affiliations:** 1 Rare Tumors and Melanoma Unit, IRCCS Istituto Tumori “Giovanni Paolo II”, Viale Orazio Flacco 65, 70124 Bari, Italy; 2 Pathology Department, IRCCS Istituto Tumori “Giovanni Paolo II”, Bari, Italy; 3 Hematology Unit, IRCCS Istituto Tumori “Giovanni Paolo II”, Bari, Italy; 4 Radiology Department, IRCCS Istituto Tumori “Giovanni Paolo II”, Bari, Italy; 5 Department of Thoracic Surgery, IRCCS Istituto Tumori “Giovanni Paolo II”, Bari, Italy

**Keywords:** Check-point inhibitor, Interstitial pneumonitis, Immune-toxicity, Melanoma

## Abstract

**Background:**

The diagnosis of check-point inhibitor-related pneumonitis (CIP) relies on radiological and clinical patterns which are not specific and can mimic other conditions (cancer progression, infectious diseases or interstitial pneumonitis). Cell pattern analysis of bronchoalveolar lavage (BAL) is well-known to support the diagnosis of interstitial lung disease; nevertheless, this analysis is somewhat performed and not required by immune-toxicity management guidelines for CIP.

**Methods:**

We performed BAL analysis in 5 metastatic melanoma (MM) patients who developed CIP among 112 patients treated with checkpoint inhibitors. We also correlated the BAL features with the computed tomography (CT) scan patterns and with various peripheral blood parameters to better define the profile of this patient population.

**Results:**

BAL flow cytometer and cytopathology analyses showed typical and homogeneous features with increased lymphoid population, prevalent CD8 + T cells and inversion of the CD4/CD8 ratio. Moreover, the extent of activated CD3 + HLA-DR + T cells was related to the grading of adverse events. Blood leucocytosis, hypoxemia, normal values for procalcitonin and lactate dehydrogenase were also found together with a cryptogenic organizing pneumonia-like radiologic pattern. In all our patients, CIP was associated with partial or complete response.

**Conclusions:**

Identification of a specific BAL cellular pattern allows clinicians to place this investigation in the appropriate position of CIP diagnosis and management to avoid misdiagnosis or considering this condition as progressive disease and delaying proper treatment.

## Introduction

In recent years, a rapidly increasing incidence of immune-related pneumonitis has been reported following the broad use of check-point inhibitors in a wide range of neoplasms both in the advanced disease and adjuvant/neoadjuvant setting [1]. Although its estimated overall incidence is less than 5% as described in clinical trials and pooled analysis [2, 3], check-point inhibitor-related pneumonitis (CIP) is being reported more frequently in the real-world setting [4, 5]. CIP is among the most severe immune related adverse events (irAEs), particularly because of its challenging diagnosis. Diagnosis is difficult because of the variable onset of CIP based on its clinical, radiological, and pathological features [6–8]. There are no specific predictive or diagnostic biomarkers for lung irAEs, making clinical symptoms and chest computed tomography scanning critical supporting a CIP diagnosis [4–6]. However, these radiological and clinical patterns are not specific and may mimic other conditions such as tumour progression showing a similar shape as lymphangitic carcinomatosis, infectious pneu-monia related to viruses such as coronavirus disease 2019 or atypical bacteria, and chemotherapy- or radiotherapy-induced interstitial lung inflammation. Although some thoracic societies [9] have suggested that cell pattern analysis of bronchoalveolar lavage (BAL) is useful for supporting the diagnosis of interstitial lung disease, this analysis has poorly described in previous reports of CIP and is not suggested by immune-toxicity management guidelines. Particularly, the prediction that check-point inhibitors act by promoting the activation and proliferation of CD8 + T cells suggests that BAL cellular analysis can provide diagnostic clues for irAE by revealing the prevalence of specific immune cells as well as changes in the CD4 +/CD8 + ratio.

We evaluated cases of CIP in five consecutive patients with stage IV melanoma treated with PD1 inhibitors alone or in combination with CTLA4 blockade. We also correlated the BAL features with the computed tomography (CT) scan patterns and with various peripheral blood parameters to better define the profile of this patient population and distinguish CIP from other forms of interstitial lung disease.

## Materials and Methods

We conducted a single-center, observational study by recruiting patients with stage IV melanoma and treated with PD-1 inhibitors (nivolumab or pembrolizumab) alone (4) or in combination with anti-CTLA4 ipilimumab (1) and who developed respiratory symptoms (worsening dyspnea, dry cough, fatigue) and signs (crackles or/and bronchial breath sounds and/or oxygen saturation below 93% at rest) and therefore underwent chest CT scanning for suspected CIP. Recruited patients underwent until a week to bronchoscopy with BAL analysis to determine the differential cell count, microbiological and cytopathology analyses. Moreover, comprehensive peripherical blood tests by gas analysis, complete white blood cells count, procalcitonin and lactate dehydrogenase (LDH) dosages were performed.

The findings of CT scanning were labelled according to the standard classification of the American Thoracic Society/European Respiratory Society (ATS/ERS) [10] as previous several reports established [6–8]. CT scan was also done to follow up the course of the adverse event.

BAL was performed during a flexible fiberoptic bronchoscopy procedure. The patient was in the wedge position and 180 mL of normal saline at 37 °C was instilled in the middle lobar bronchus in three boluses. Next, 50 mL of BAL was gently recovered and collected into sterile bottles. The recovered BAL fluid was filtered through gauze and the samples were processed within 1 h of collection. One hundred microliters of the sample were cytocentrifuged, and then smeared and stained with May-Grünwald Giemsa. For differential cell counting, a sample corresponding to 250,000–300,000 cells were passed through a multipore filter (0.22-µm). After staining, a minimum of 2 × 100 cells was counted.

The total cell number was assessed in a Neubauer chamber. The number of cells obtained ranged from 1.3 × 10^5^ to 2.5 × 10^5^ for millilitres. Depending on the number of cells obtained, we stained 1–3 × 10^5^ cells in 100 µL of phosphate-buffered saline (PBS; Oxoid, Hampshire, England) with the following monoclonal antibodies combination: one tube with BD Multitest™ 6-Color TBNK Reagent (BD Biosciences, San Jose, CA, USA): CD3 FITC clone SK7, CD16 PE clone B73.1, CD56 PE clone NCAM16.2, CD45 PerCPCy™5.5 clone 2D1, CD4 PE-Cy™7 clone SK3, CD19 APC clone SJ25C1, and CD8 APC-Cy™7 clone SK1; another tube with BD CD45 FITC clone 2D1, CD3 PerCPCy™5.5 clone SK7, CD4 PE-Cy™7 clone SK3, CD8 APC-Cy™7 clone SK1, and anti-human HLA-DR APC clone G46-6. The samples were incubated with antibodies at room temperature in the dark for 30 min. Lysing solution (BD Biosciences) was added and the sample was incubated for 5 min. The samples were washed in PBS, decanted again, and resuspended in 500 µL of PBS. Cells were acquired in a previously set up FACSCanto II cytometer. Data analysis was performed with the Beckman Coulter analysis software Kaluza (Brea, CA, USA).

## Results

### Clinical features

Between 2018 and 2019 we identified 5 consecutive cases of suspected CIP among 112 patients with stage IV melanoma treated with checkpoint inhibitors. The detailed disease, clinical and therapeutic features of patients and of their irAEs are shown in Table 1. Comparative features of the cohort of patients who did not developed CIP were reported in Table 2. Although the relevant difference in size between the two groups, there were similar prevalence of chronic obstructive pulmonary disease (COPD) and lung involvement which were identified as CIP risk factors. Conversely a former or current smoker status was more common in patients with CIP while the BRAF V600 was more frequent in patient without CIP. In our series the time onset of the respiratory irAE was variable ranging from 8 to 88 weeks with common clinical features being dyspnoea as the relevant symptom that was graduated as G4 in two cases. All patients recovered from the effects of toxicity after steroid treatment for a median of 2 months. PD1 inhibitors were permanently discontinued in 4 patients, whereas one patient was re-started on treatment until disease progression which happened 6 months later. In the two patients who reported the highest grade of severity, we observed a recurrence after the completion of steroid tapering. Both patients retained a response to steroid but after recovering still maintained this treatment for 6 and 8 months respectively. Interestingly, all patients showed a previous or subsequent melanoma response to checkpoint inhibitors and consequently lasting progression free survival (PFS) and overall survival (OS).

**Table 1:** Patient features

	Patient 1	Patient 2	Patient 3	Patient 4	Patient 5	Normal range
Patient’s features						
Age (years)	52	77	58	75	43	
Sex	Male	Female	Female	Male	Male	
Smoker status	Current	Former	Former	Former	Never smoke	
Comorbidity	Atrial fibrillation	Diabetes, hypertension, copd	Diabetes, hypertension, obesity	None	None	
BRAF status	Wild type	Wild type	Wild type	Wild type	V600E	
Melanoma type	Unknown origin	Cutaneous	Cutaneous	Unknown origin	Cutaneous	
M stage^a^	M1d	M1a	M1b	M1b	M1a	
Tumor involvment	Lymphnodes, lung, brain	Soft tissues, lymphnodes	Soft tissues, lung	Lung	Sof tissue	
Treatment features						
Treatment regimen	Nivolumab as 1° line	Pembrolizumab as 1° line	Pembrolizumab as 2° line	Nivolumab as 1° line	Ipilimumab plus nivolumab as 1° line	
Best response	Partial response	Partial response	Partial response	Partial response	Partial response	
Progression-free survival, months^b^	12	24+	43+	26	8+	
OS, months^c^	14+	24+	84+	36+	8+	
Clinical features						
Onset (weeks)	8	44	88	60	6	
Clinical symptoms	dyspnoea, fatigue	dyspnoea, dry cough, fatigue	dyspnoea, fever, fatigue	dyspnoea, fatigue	dry cough, dyspnoea	
Grading irAE	G3	G4	G4	G3	G2	
Outcomes	Recovered	Subsequent recurrences	Subsequent recurrences	Recovered	Recovered	
Not lung toxicites	Skin (vitiligo)	Skin (vitiligo)	None	None	Gatrointestinal (colitis)	
Blood tests						
PCO_2_ mmHg	31	44	50	58	44	32–48
PO_2_ mmHg	41	69	71	61	88	83–108
WBC (x 10^3^/μL)	17,2	13,4	11,8	16,9	7,8	4–10
Neutrophils	11,4	8,5	9,6	11,2	5,07	1,7 –7,6
Lymphocytes	4,8	4,06	1,3	5,2	2,1	1–3,2
N/L	2,38	2,11	7,21	2,15	2,39	
LDH	>ULN	<ULN	<ULN	<ULN	<ULN	
Procalcitonin (ng/ml)	0,34	0,5	3,6	1,2	0,2	<2
CT scan						
CT scan pattern	NISP	COP	COP	NISP	COP	
Lung involvement	Upper and lower lobes	Upper and lower lobes	Mainly lower lobes	Upper and lower lobes	Mainly upper lobes	

Detailed patient, treatment, clinical, blood and CT scan features of five patients with check-point inhibitor-induced pneumonitis. ^a^M stage assessed according the 8th edition of AJCC melanoma staging system; ^b^ + means ongoing; ^c^ + means alive

**Table 2:** Melanoma cohort

Cohort population (112)	CIP group 4% (5)	No CIP group 96% (107)
Median age	58	60
Male sex	60%	51%
Former or current smoker status	80%	35%
COPD	20%	18%
BRAF V600	20%	46%
Lung involvement	60%	49%
PD1 inhibitors as treatment regimen	80%	76%
ORR	100%	38%

Comparative clinical, therapeutic features of the analyzed cohort of 112 patients treated with checkpoint inhibitors among with there were 5 cases of CIP

### Blood and scan features

**Fig. 1:**
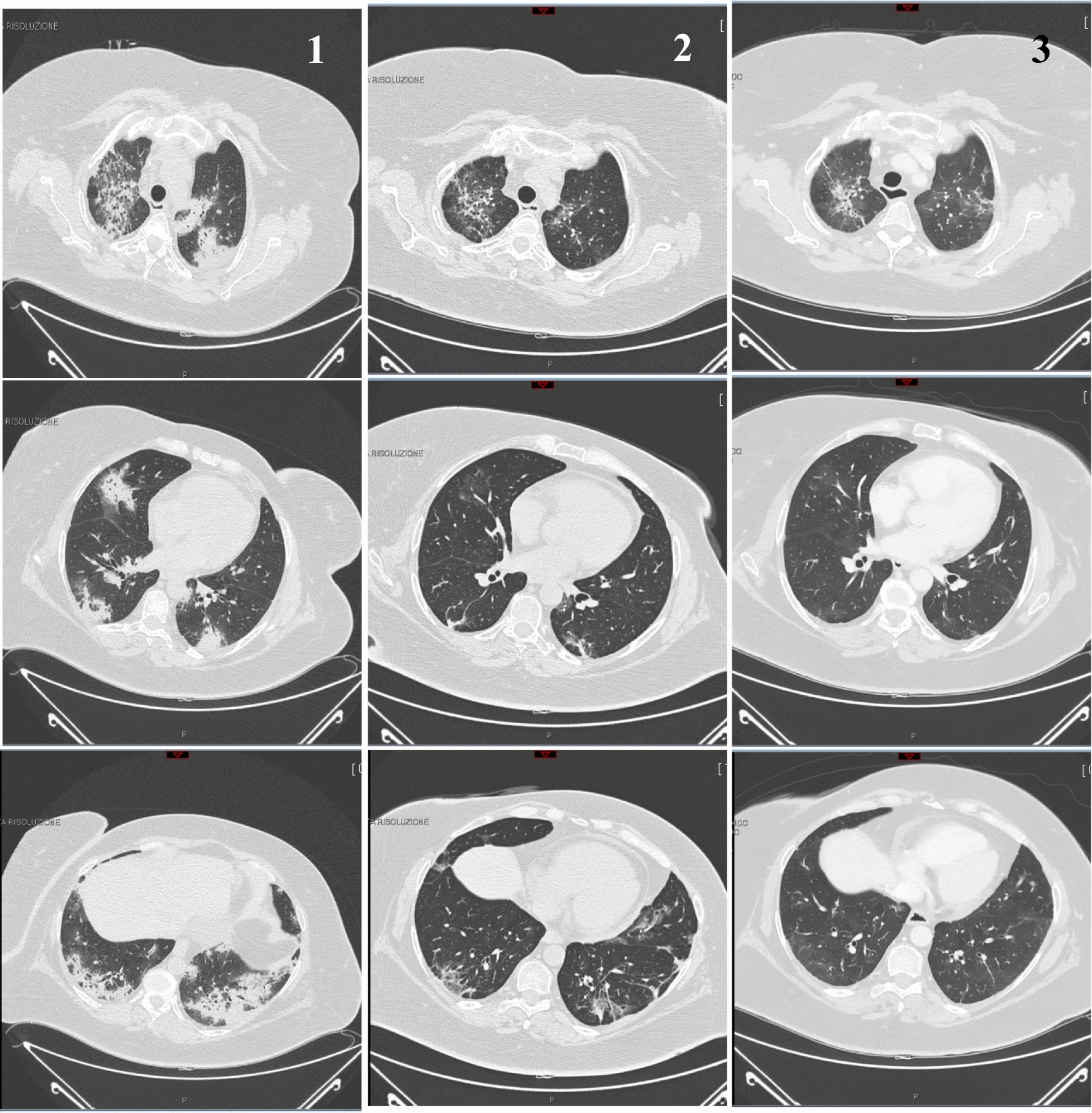
Radiological and cytological features in a representative patient. **A**. Chest computed tomography at different timepoints. 1. CT images at admission (at 22 months after starting pembrolizumab), showing a COP pattern with multiple pseudo-nodular parenchymal consolidations with irregular and shaded margins, spread bilaterally but more extended to the lower lobes. Ground glass areas were observed in the upper right lobe. 2. CT image at 1 month after the onset of CIP and after i.v. methylprednisolone, showing significant resolution of the bilateral consolidations with minimal resolution of ground-glass opacities. 3. CT image at 4 months after discharge showing further improvement in radiological alterations

The gas analysis showed common and not specific features with variable degrees of hypoxemia and hypercapnia related to the radiological spread. Among the blood tests, leucocytosis with prevalent neutrophilia was found in 4 out of 5 patients while both inflammatory indices as procalcitonin and tumour markers like LDH appeared within the normal limits in all cases except one. Likewise, the radiological pattern appeared not specific with 2 non-specific interstitial pneumonia (NISP) appearances and 3 cryptogenic organizing pneumonia (COP) (Fig. 1) along with a variable range of lung involvement.

### BAL features

The number of cells obtained from the BAL was 1.3–2.5 × 10^5^ for millilitres of fluid. BAL cellular analysis revealed typical and homogeneous features with increased lymphoid population which was observed along with relevant enrichment of CD8 + T cells and consequent inversion of the CD4/CD8 ratio (Table 3). Macrophages, which normally represents the primary component of BAL, were found in the lower limit of the norm, while the rate of neutrophils, eosinophil, natural killer cells and B cells were within the normal range. Finally, we found a relevant rate of activated CD3 + HLA-DR + T cells ranging from 13 to 36% that seemed to be related to the grading of adverse events as well as to recurrence risk of this irAE. No other correlation was found with blood parameters or radiologic patterns such as NSIP or COP.

**Table 3:** BAL parameters

	Patient 1	Patient 2	Patient 3	Patient 4	Patient 5	Normal range
Total cells (x 10^5^/ml)	1,8	1,8	2,5	1,5	1,3	
Macrophages	78%	80%	77%	72%	66%	75–85%
Neutrophils	0	0	5%	3%	2%	1–2%
Lymphocytes	22%	20%	26%	24%	30%	8–12%
Eosinophils	0	0	2%	1%	2%	0–0,5%
T CD3+	99%	95.8%	96%	95%	93%	70–90%
T CD4+	35%	17.2%	41%	38%	39%	35–45%
T CD8+	60.3%	77%	52%	47%	50%	30–40%
Natural killer CD3-CD16 + CD56+	0,70%	2,60%	3%	2%	3%	1–7%
B CD19+	0	0,50%	1%	1%	1%	0–7%
CD4/CD8 RATIO	0.6	0,2	0,7	0,8	0,7	0,8–2
CD3 + HLA-DR+	25.8%	36%	31%	24%	13%	

Flow cytometer of BAL in 5 melanoma patients with CIP

**Fig. 2:**
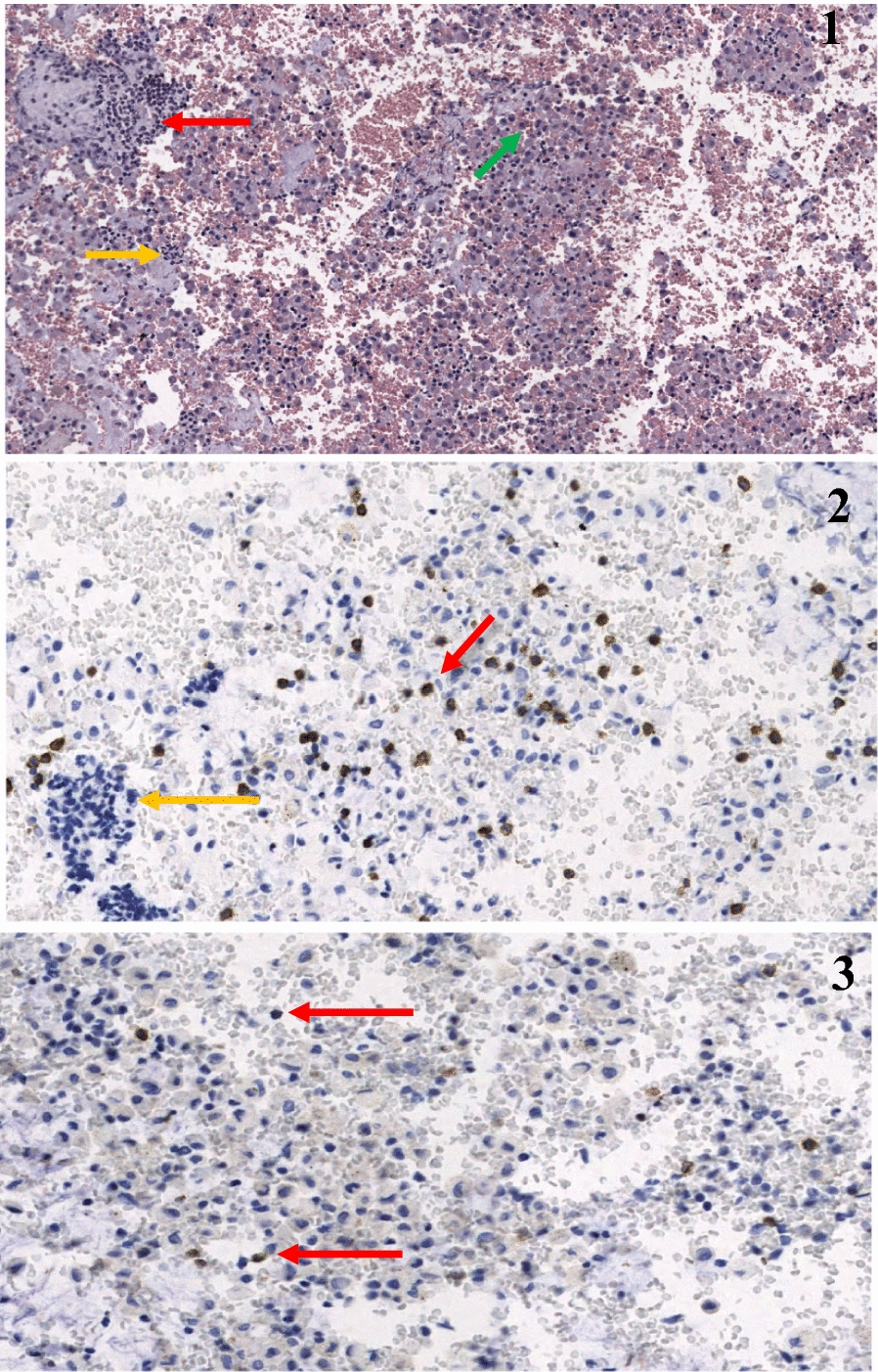
**B** Cytologic pattern of BAL specimens. 1. Bronchoalveolar wash stained with hematoxylin–eosin (20x): red cells, mucus, normal bronchial epithelia (yellow arrow) on a carpet of histocytes (green arrow), and lymphocytes (red arrow); 2. Immunocytochemistry staining of CD8 lymphocytes (red arrow) (normal bronchial epithelia, yellow arrow) (40x); 3. Immunocytochemistry staining of CD4 lymphocytes which appeared less represented (red arrows) (40x)

BAL cytopathology (Fig. 2) confirmed flow cytometry analysis and did not reveal melanoma cells, indicating the absence of disease progression while BAL fluid culture ruled out bacterial or fungal infections.

## Discussion

Among irAEs, distinguishing lung toxicity remains difficult because its diagnosis is based on exclusion and often missed [4], as no clear statement has been developed to aid in its definition. In addition to pooled meta-analyses which have evaluated the incidence and risk factors of lung irAEs [2–5], there are several single case reports or small case series as well as three main retrospective studies focusing on diagnostic issues [6–8]. These reports emphasized the use of thoracic scan imaging and proper clinical assessment as the main diagnostic tools. They adopted the ATS/ERS classification to standardize CIP evaluation and showed that COP pattern was the most frequent radiological feature of this lung irAE as observed in our patients (Fig. 1). However, no specific radiological features have emerged as pathognomonic of CIP in these reports. Alternatively, using BAL as a diagnostic tool has poorly investigated and has mostly been used in CIP to rule out infection or cancer aetiology. Conversely, the immune cell pattern of BAL was elegantly investigated by Suresh et al. to gain insight on CIP pathogenesis [11]. By matching the BAL samples of patients with and without CIP, they argued that this lung irAE was a dysregulated inflammatory response involving an inhibition of tolerogenic T regulatory cells and a boost of proinflammatory lymphocytic and myeloid subsets [11]. In our series, we systematically reviewed the results of cellular analysis of BAL to define a pattern indicative of CIP. In all cases, the same pattern emerged, characterized by a T lymphocytosis with CD8 + counts higher than normal values and thus an inverted CD4/CD8 ratio. Thus, we could confirm the previous biologic data about the CD4 deficiency as the main indicator of CIP as well as the T lymphocytosis. This evidence could strengthen the search for a therapy in the direction of drugs already used in this setting as showed with the successful adoption of synthetic vasoactive intestinal peptide in a melanoma patient with CIP [12]. Moreover, in a homogeneous small melanoma population of CIP we showed a correlation between the rate of activated T cells and the severity of adverse events that could be also used as a marker to early monitor treatment efficacy as well as of recurrence risk. Thus, these data were consistent with the ability of BAL fluid to uncover immune events involving T-cells at the interstitial and alveolus as also showed by Tanaka [13]. Indeed, sequencing of the T cell receptor revealed overlap between the complementarity-determining region of BAL CD3 + cells and tumor-infiltrating lymphocytes (TILs) from the same patient who developed CIP during treatment with nivolumab for stage IV kidney cancer [13]. Similarly, BAL CD8 + cells and TILs from this patient exhibited the same expression pattern of PD1 and T cell immunoglobulin mucin-3 receptor which differed from that of BAL CD8 + cells found in bacterial or chemotherapy-induced pneumonitis [13]. Therefore, BAL findings may also explain the occurrence of CIP in cancers that respond to PD1 inhibitors as we observed in our patients and has been broadly reported [9, 14].

Overall, BAL features of CIP could distinguish this interstitial pneumonitis from other immune or drugs-mediated forms. Although lymphocytosis is a common denominator of interstitial pneumonitis with COP patterns such as sarcoidosis [15–18], in CIP there are no increase of neutrophil and macrophage relative number as well as no evidence of foamy macrophage which are typical of these connective lung disease [17, 18]. Moreover, autoimmune pneumonitis with NSIP pattern like idiopathic pulmonary fibrosis are often associated with paucity of lymphocyte in BAL [18] instead of CIP in which we do not observe difference of features regardless of radiological pattern. Finally, we observed in CIP the absence of eosinophil-dominant BAL typical of drug-mediated forms which often resemble hypersensivity pneumonitis [19, 20].

In our series, BAL lymphocytosis did not correlate with blood gas features and did not match lymphocytosis in the peripheral blood even though leucocytosis was observed in four of the five cases. No previous studies have described the behaviour of circulating cells during CIP or showed that procalcitonin levels were within normal ranges, which also allowed us to rule out infections which was confirmed by BAL fluid culture.

In conclusion our comprehensive study involving flow cytometry analysis offers a clear diagnostic tool by showing that the BAL T cell population has a distinctive pattern.

Some limitations of our study deserve to be underlined. Indeed, we evaluated few patients with only melanoma as cancer type and with very good response to check-point inhibitor therapy. In the diagnostic work-up of CIP, beyond contraindications in patient with cardio-respiratory instability, the usefulness of BAL is controversial because of concerns about the absence of prospective studies which could weight its diagnostic contribution in patients with different tumour type, variable grading of irAEs and responses to therapy.

Similarly, we were unable to determine the prognostic value of the immune features observed in BAL analysis even if the role of CD3 ± HLA-DR ± cells is noteworthy to further investigate. Further investigations are also needed to identify inherited host or tumour genetic features that can predict lung irAEs as well as of circulating markers for early detection or narrowing of the CIP diagnosis.

## Conclusion

In summary, identification of a specific BAL cellular pattern allows clinicians to place this investigation in the appropriate position of CIP diagnosis and management to avoid a misdiagnosis or considering this irAE as progressive disease and delaying proper treatment.

## Data Availability

All data generated or analyzed during this study are included in this published article.

## Abbreviations

CIP: Checkpoint inhibitor-related pneumonitis
BAL: Bronchoalveolar lavage
PD1: Programmed cell death protein 1 receptor
CD8: Cluster of differentiation 8
CD4: Cluster of differentiation 4
LDH: Lactate dehydrogenase
COP: Cryptogenic organizing pneumonia-like
irAE: Immune-related adverse event
CTLA4: Cytotoxic T-lymphocyte antigen 4 receptor
CT: Computed tomography
ATS/ERS: American Thoracic Society/European Respiratory Society
